# High‐Efficient and Dosage‐Controllable Intracellular Cargo Delivery through Electrochemical Metal–Organic Hybrid Nanogates

**DOI:** 10.1002/smsc.202100069

**Published:** 2021-09-09

**Authors:** Bowen Zhang, Dinuo Zheng, Shi Yiming, Kazuhiro Oyama, Masahiro Ito, Masaomi Ikari, Takanori Kigawa, Tsutomu Mikawa, Takeo Miyake

**Affiliations:** ^1^ Graduate School of Information, Production and Systems Waseda University Kitakyushu Fukuoka 808-0135 Japan; ^2^ RIKEN Center for Biosystems Dynamics Research 1-7-22 Suehiro-cho, Tsurumi-ku Yokohama Kanagawa 230-0045 Japan; ^3^ PRESTO Japan Science and Technology Agency 4-1-8 Honcho Kawaguchi Saitama 332-0012 Japan

**Keywords:** cell viabilities, intracellular cargo delivery rates, intracellular deliveries, membranes, metal–organic hybrid materials

## Abstract

Hollow nanostructures combined with electroporation are potentially valuable in interdisciplinary fields due to their ability to transport versatile cargos into adhesive cells. However, they require voltages over 1.5 V to electroporate the physical barrier of the cell membrane inducing cell death and differentiation processes. Intracellular delivery is exhibited using a metal–organic hybrid nanotube (NT) stamp that physically inserts into the cells and subsequently injects versatile molecules at an extremely low voltage of ±50 mV (less than membrane potential). The hybrid NTs consist of Au NTs polymerizing electrochemically 3,4‐ethylenedioxythiophene monomer and supportive polycarbonate membrane. The hybrid stamp improves the cell viability by 94% for a 30 min physical insertion while decreasing the cell viability to 1% using the original Au NTs. Furthermore, the hybrid stamp acts as an electrochemical gate that can open the pore at ±50 mV to transport small molecules of calcein dye with high efficiency (99%) and viability (96.8%). The hybrid nanogate can also transport large molecules of green fluorescent protein (GFP) with 84% efficiency and 98.5% viability, and GFP plasmid at a transfection rate of ≈10%. Thus, the present hybrid stamping can potentially deliver versatile molecules into adhesive cells.

## Graphical abstract

The hybrid nanotube stamp improves the cell viability by 94% for 30 min physical insertion. Furthermore, the hybrid stamp acts as an electrochemical gate that can open the pore at ±50 mV to transport small molecules of calcein dye with high efficiency (99%) and high viability (96.8%).

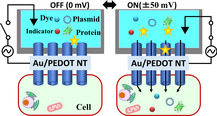

## 1 Introduction

Intracellular cargo delivery plays an important role in fundamental biological research^[^ ^1^ ^]^ and therapeutic medical applications,^[^ ^2^ ^]^ ranging from intracellular function analysis,^[^ ^3^ ^]^ gene encoding for cellular reprogramming,^[^ ^4^ ^]^ and the inhibition of gene expression inside cells.^[^ ^5^ ^]^ Cargo delivery requires safe and efficient access to cells and different intracellular locations such as the nucleus and mitochondria due to impermeable outer cell membranes, including small molecules,^[^ ^6^ ^]^ nucleic acid genes,^[^ ^7^ ^]^ amino acid proteins,^[^ ^8^ ^]^ nanosensors,^[^ ^9^ ^]^ and organelles.^[^ ^10^ ^]^

Nanostructures are promising candidates for membrane disruption where versatile cargos transport into cells to overcome the impermeable plasma membrane. Silicon nanowires,^[^ ^11^ ^]^ diamond nanoneedles,^[^ ^12^ ^]^ carbon nanofibers,^[^ ^13^ ^]^ and ZnO nanowires^[^ ^14^ ^]^ have been developed for mechanical needle penetration into living cells for molecular delivery. Although they improved the delivery efficiency, but the cargo delivery is still restricted by some limitations such as small amount of the loaded/released molecules to/from the nanostructure surfaces (dosage/dosage control). Consequently, hollow nanostructures, including silicon,^[^ ^15^ ^]^ carbon,^[^ ^16^ ^]^ and Al_2_O_3_^[^ ^17^ ^]^ nanotubes (NTs), spontaneously penetrated cells with connection to microfluidic channels, which can control molecular flow and subsequent direct delivery into cells through nanostructured ducts. A plasmid DNA,^[^ ^18^ ^]^ Ca^2+^ indicator,^[^ ^19^ ^]^ fluorescent dye,^[^ ^20^ ^]^ quantum dot,^[^ ^21^ ^]^ and protein^[^ ^22^ ^]^ were delivered into different cell types using the aforementioned methods. For further improvement, the NTs were combined with external forces such as mechanical,^[^ ^18^ ^]^ electrical,^[^ ^23^ ^]^ and photothermal poration.^[^ 24, 25 ^]^ Among these methods, an NT‐electroporation platform is an excellent technique for intracellular delivery, providing improvement in delivery efficiency and dosage controllability. However, the cell viability still remained a problem due to the high voltages requirement of over 1.5 V to create transient pores in the plasma membrane.^[^ ^26^ ^]^ Furthermore, such high electrical voltage induces problematic intracellular signaling, which is relative to differentiation^[^ ^27^ ^]^ and reprogramming.^[^ ^28^ ^]^

**Figure 1:**
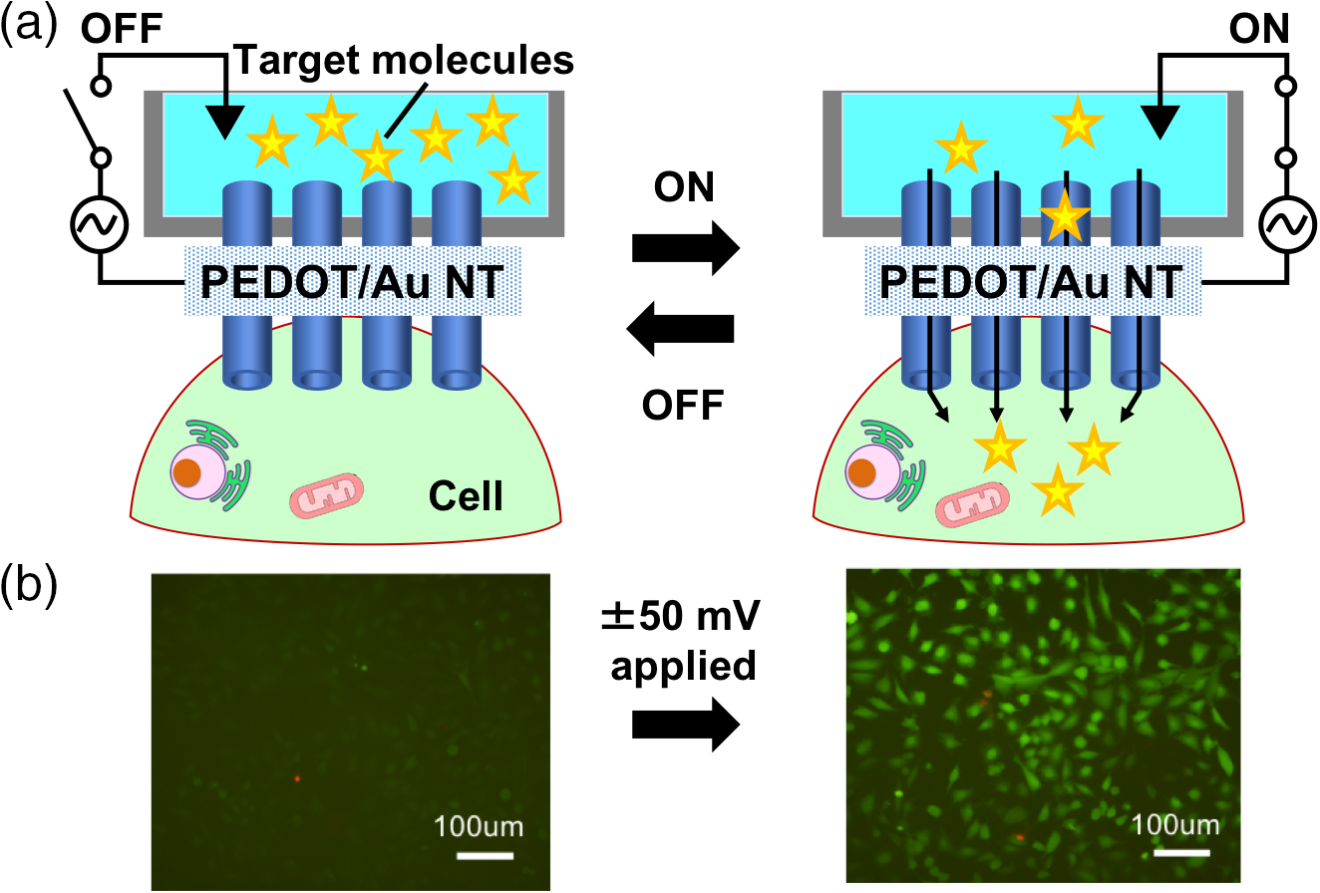
Electrochemical PEDOT/Au hybrid NT stamping for intracellular delivery. a) The hybrid NTs can penetrate the adhesive cells and then inject the target molecules when we apply the external voltage to the NT stamp. b) Fluorescence images of calcein‐delivered cells with and without the voltage.

Here, we develop metal–organic hybrid NTs that can be inserted into adhesive cells and subsequently deliver versatile molecules of calcein dye, green fluorescent protein (GFP) protein, and plasmid DNA into different types of adhesive cells with precise dosage control (**Figure** **1**). The hybrid NTs consist of Au NTs electrochemically polymerizing a 3,4‐ethylenedioxythiophene (EDOT) and supportive polycarbonate membrane. Polymerized EDOT (PEDOT) has been widely used in the field of bioelectronics.^[^ ^29^ ^]^ In our previous studies,^[^ 30, 31 ^]^ we characterized the electrochemical behavior of PEDOT and hydrogel composite films as well as employed them for the electrochemical stimulation of C2C12 myotubes and iontophoretic drug delivery patches through human skin and animal skin. Here, we characterize the molecular flow through the PEDOT‐coated hybrid NTs with and without the applied potentials. Without the potential, the PEDOT‐layer‐coated inner NT surface expanded due to the blockage of molecular flow through the hybrid NTs. When we applied an alternating current (AC) voltage of ±50 mV to the hybrid NTs, the volume of the PEDOT layer repeatedly changed because of the doping and dedoping process, which opened the gate to inject molecules into the cells. Furthermore, we measured the asymmetric structures of the hybrid NTs, which have a small‐diameter needle tip opening at one face and a large‐diameter base opening at the other, and such a unique structure causes the electroosmotic flow to deliver the molecules slowly in the base‐to‐tip direction. This is a novel architecture and delivering system compared with those reported in previous works^[^ 1, 2 ^]^ with NT combination that allow a direct delivery of versatile cargos into cells.

## 2 Results and Discussion

### 2.1 Fabrication of PEDOT/Au Hybrid NTs

**Figure 2:**
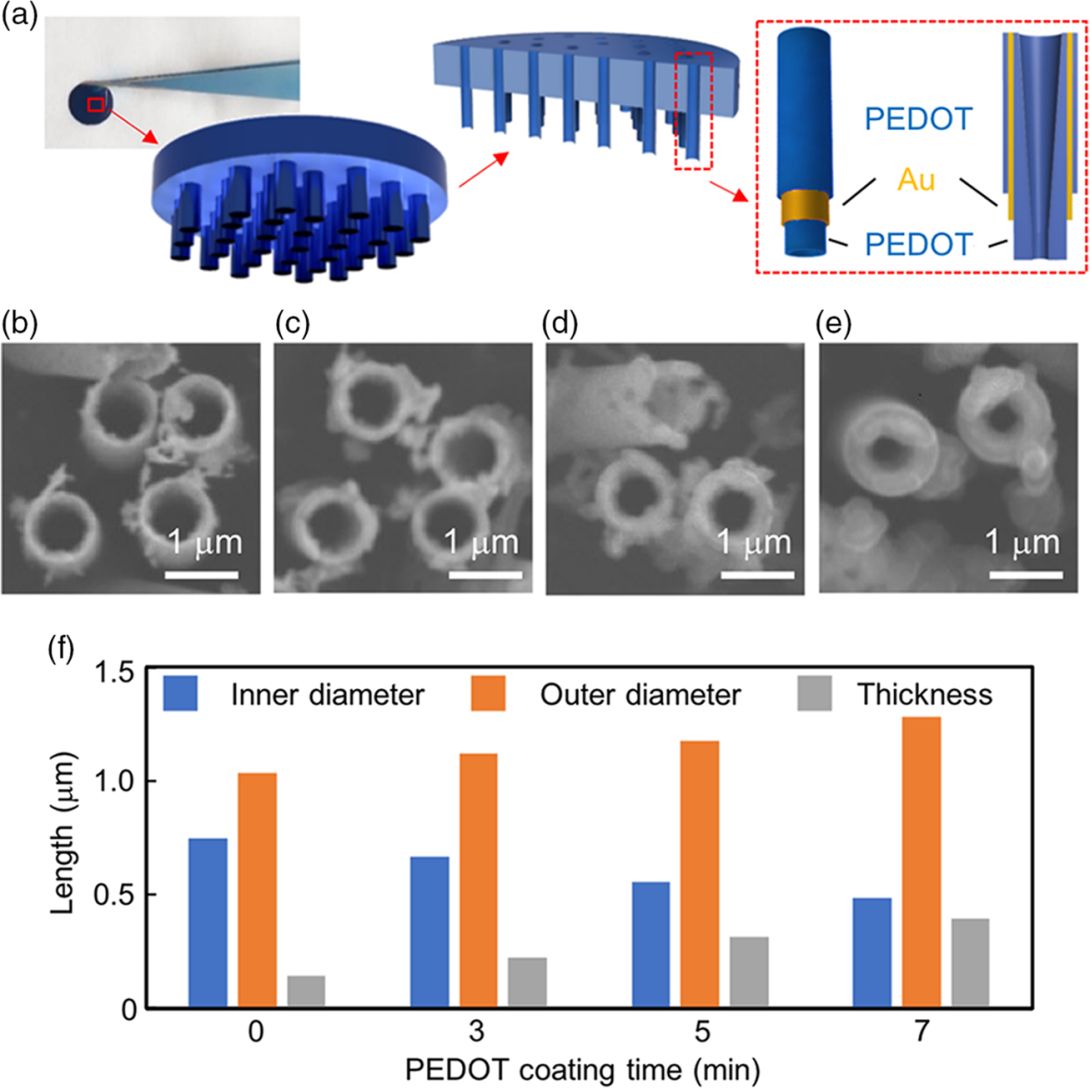
PEDOT/Au NT structures. a) Picture and schematics of PEDOT/Au NT membrane. b–e) SEM images of Au NTs at the different coating times of PEDOT film at b) 0 min, c) 3 min, d) 5 min, and e) 7 min. f) Summarized SEM results of the PEDOT/Au NTs in inner diameter, outer diameter, and wall thickness.

The PEDOT/Au hybrid NTs are fabricated through a three‐step process: 1) electroless Au plating on tracked‐etched polycarbonate (TEPC) template, 2) wet and dry etching of the top surface, and 3) electrochemical polymerization of PEDOT nanolayer on the electroplated Au NT surface. The preparation of the Au NT membrane and related material characterizations are generally followed as per our previous work.^[^ ^32^ ^]^ For electroless Au plating, a tin–palladium metallic catalyst layer is formed on the TEPC surface. Then, the membrane is immersed in a gold plating solution to produce the Au/TEPC membrane. The top surface of the Au/TEPC membrane was reacted with aqua regia for removing Au nanolayer and subsequent O_2_ plasma for etching TEPC that we refer to as Au NT/TEPC. Next, we applied 1 V to the Au NT/TEPC membrane in a mixed solution including EDOT monomer and LiClO_4_ dopant to polymerize the PEDOT nanolayer on Au NTs. Afterward, the original color of Au NTs is changed to the blue of the PEDOT/Au hybrid NT surface (**Figure** **2**a). The wall thickness of the hybrid NT was increased by lengthening the polymerization time of PEDOT (Figure 2b–f). Moreover, the original inner diameter of 750 nm gold NT decreased to 670 nm at 3 min, 550 nm at 5 min, and 485 nm at 7 min PEDOT coating when measured from the scanning electron microscopy (SEM) images (Figure 2b–e). Conversely, the inner diameter of the opposite surface decreased slightly to 621 nm at 3 min, 620 nm at 5 min, and 613 nm at 7 min (Figure S1, Supporting Information). The dense PEDOT layer was formed only on the tip of Au NTs, resulting in the asymmetric nanostructures of PEDOT/Au NTs on the top and bottom of the membrane. These results indicate that we succeeded in producing metal‐organic hybrid NT arrays with tunable nanostructures.

### 2.2 Molecular Flow through the Hybrid NTs

**Figure 3:**
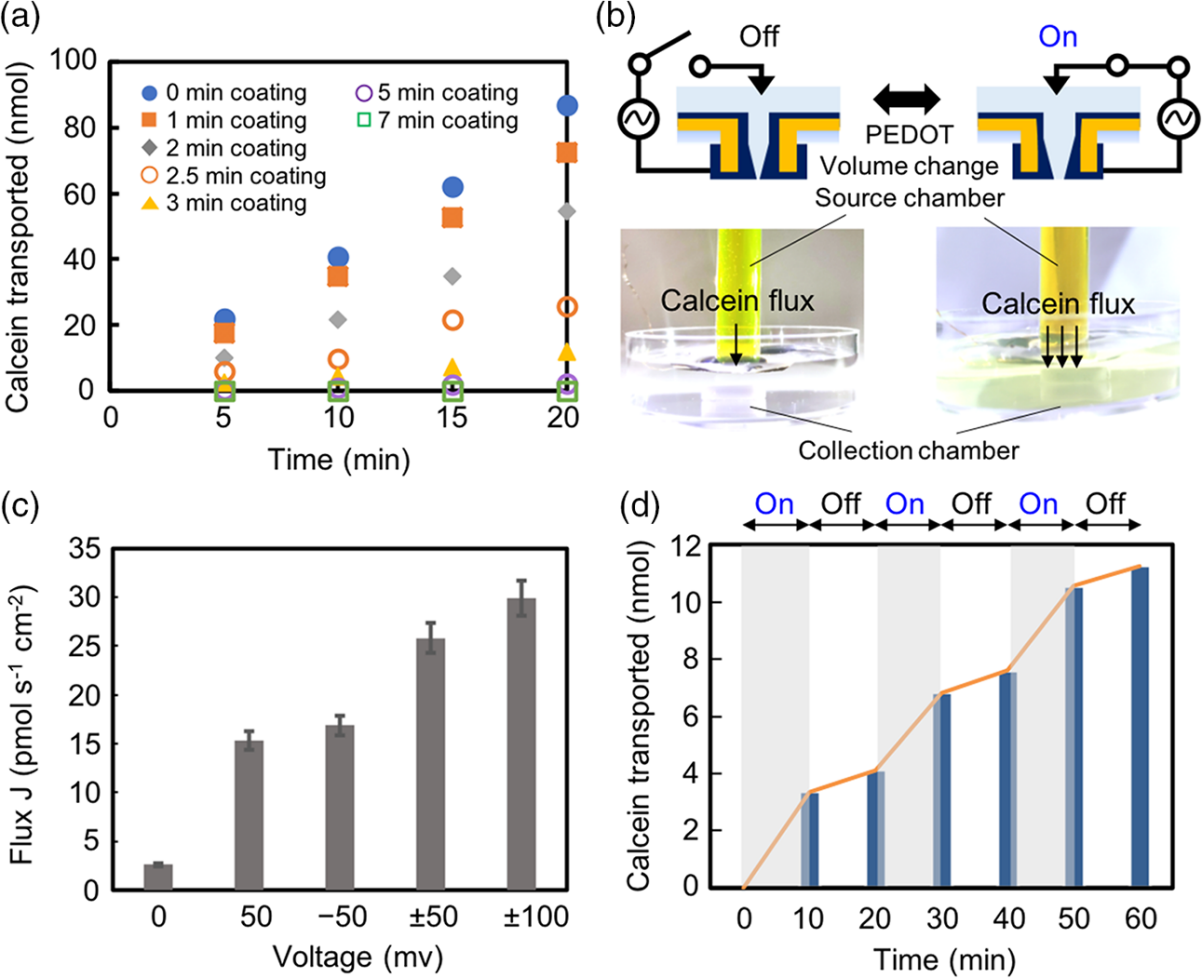
Calcein flow through the hybrid membrane. a) Amount of transported calcein with the different PEDOT coating times. b) Pictures and schematics of calcein flow with and without voltage supply. c) Calcein flux through PEDOT‐5/Au NTs at the different voltages. *n* = 3. d) Amount of transported calcein through PEDOT‐5/Au NTs when we applied a sequential voltage of ±50 mV and 0 V for three cycles.

The molecular flow through the PEDOT/Au hybrid NT arrays was investigated with fluorescence measurements from the transported calcein dye described in our previous paper using Au NT membrane.^[^ ^32^ ^]^ We made a needle stamp for the source chamber to measure the amount of transported calcein through the NT membrane. The stamp consisted of the hybrid membrane (diameter: 8 mm) of PEDOT/Au NTs at different coating times and 10 mm PBS solutions, including 1.6 mm calcein molecules in a glass tube. When the stamp contacts with the collection chamber in the stirring phosphate buffered saline (PBS) solution, calcein molecules diffuse from the highly concentrated source chamber to the collection chamber through the PEDOT/Au hybrid NT membrane. The calcein flux is defined as $J=DC(\pi r^{2}n/\pi R^{2})/l$,^[^ ^32^ ^]^ where *D* is the diffusion coefficient (7.7 × 10^−14^ cm^−2^ s^−1^ for the calcein molecule),^[^ ^33^ ^]^ *C* is the concentration of 1.6 calcein, $\pi r^{2}$ is the inner diameter of PEDOT/Au NT, *n* is number of hybrid NTs on membrane area ($\pi R^{2}$), and *l* is the membrane thickness of 25 μm. According to the equation, the calcein molecules diffuse as a function of $\pi r^{2}$. The calcein flow through the original Au NT membrane increased lineally up to 86.9 nmol for 20 min (**Figure** **3**a), resulting in *J* = 0.361 nmol s^−1^ cm^−2^. When we polymerized the PEDOT on Au NT membrane at the different times of 1, 2, 2.5, 3, 5, and 7 min, *J* was decreased to 0.303, 0.213, 0.110, 4.63 × 10^−2^, 9.82 × 10^−3^ nmol s^−1^ cm^−2^, and ≈0 nmol s^−1^ cm^−2^, respectively. This is because the inner diameter of the hybrid NTs was decreased by coating PEDOT layer (Figure 2). From the equation and the flux data in Figure 3a, we estimated the inner diameter of PEDOT‐coated hybrid NTs, being 687 nm for 1 min, 576 nm for 2 min, 414 nm for 2.5 min, 269 nm for 3 min, 123 nm for 5 min, and ≈0 nm for 7 min. The estimated inner diameters were shorter than that from SEM images (Figure 2) because the dried PEDOT layer on Au NTs expanded in the aqueous solution. When we immersed the dried PEDOT/Au hybrid microelectrode into a PBS solution, the original thickness of 14 μm was increased to 25 μm thickness hybrid electrode (Figure S2a,b, Supporting Information) due to water swelling into the PEDOT layer.

The mechanical and electrical properties of PEDOT layers on Au NTs can be controlled by the external voltages described in our previous works.^[^ ^34^ ^]^ Dopant ions of both anion and cation molecules travel between the solution and PEDOT film when applying the positive oxidation voltage *V*_ox_ to the PEDOT film.^[^ ^35^ ^]^ Thus, the oxidation current can be observed (Figure S3, Supporting Information). In contrast, dopant ions in PEDOT film release to the solution at the reduction voltage (*V*_rd_) due to the dedoping process. Such oxidation and reduction current can enhance when we increased the applied voltages (Figure S3, Supporting Information). We confirmed the different *J* values when we applied the different voltages in AC and DC modes using these doping and dedoping processes. *J* of 2.63 pmol s^−1^ cm^−2^ without the applied *V* was enhanced to 15.3 pmol s^−1^ cm^−2^ at 50 mV for 10 min and 16.9 pmol s^−1^ cm^−2^ at −50 mV for 10 min (Figure 3c). Further enhancement using AC voltage of ±50 and ±100 mV for 10 min was achieved to 25.8 and 29.9 pmol s^−1^ cm^−2^, respectively. Such enhancement was not observed when we used the Au NT membrane (Figure S4, Supporting Information). This enhancement is attributed to the following two reasons: the change in volume and surface‐charge modulation of PEDOT layer on NT surface and the electroosmotic flow (EOF) rectification effect. As shown in Figure S2c, Supporting Information, the thickness of the PEDOT layer increased at 50 mV, and the original thickness was regained at −50 mV. Such a change in volume is attributed to the doping and dedoping of both anion and cation molecules between the PEDOT and aqueous solutions that modulate the charge of the PEDOT surface. The volume‐changed and charged hybrid NTs may accelerate the molecular flow when the DC voltage is applied. In the AC mode, we infer that the EOF rectification occurred through the asymmetric nanostructured NT membrane. In general, the EOF rectification, which is the higher flow rate in one direction through the membrane than that in the opposite direction, occurs when a conical chapped nanopore membrane is used.^[^ ^36^ ^]^ Conical nanopores include a small‐diameter tip opening at one face and a large‐diameter base opening at the other. When the molecules flow through the conical membrane, the flow rate in the base‐to‐tip direction is higher than that in the tip‐to‐base direction.^[^ ^37^ ^]^ As our hybrid NT membrane has asymmetric nanostructures (Figure 2 and S1, Supporting Information), we confirmed a rectified ionic current through the hybrid NTs when a voltage ranging from −100 to 100 mV was applied (Figure S5, Supporting Information). When an AC voltage was applied to the EOF‐rectified membranes, a high base‐to‐tip flow rate was observed in one voltage half cycle, whereas a low tip‐to‐base flow rate was observed in the second half cycle as described in previous report.^[^ ^38^ ^]^ This is called as the AC EOF pump. The EOF pump at the AC voltage may accelerate molecular flow through the hybrid NTs. Importantly, when we applied the sequential voltages of ±50 mV (on state, for 10 min) and 0 V (off state, for 10 min) for three cycles, the flow of calcein through the hybrid NTs in the voltage on state was three times higher than that in the voltage off state (Figure 3d). Therefore, the proposed hybrid NTs allow the calcein flow at different flux rates when we control the thickness of the PEDOT nanolayer and act as an electrochemical nanogate to control the flow rate with an external voltage source.

### 2.3 Cell Viability

**Figure 4:**
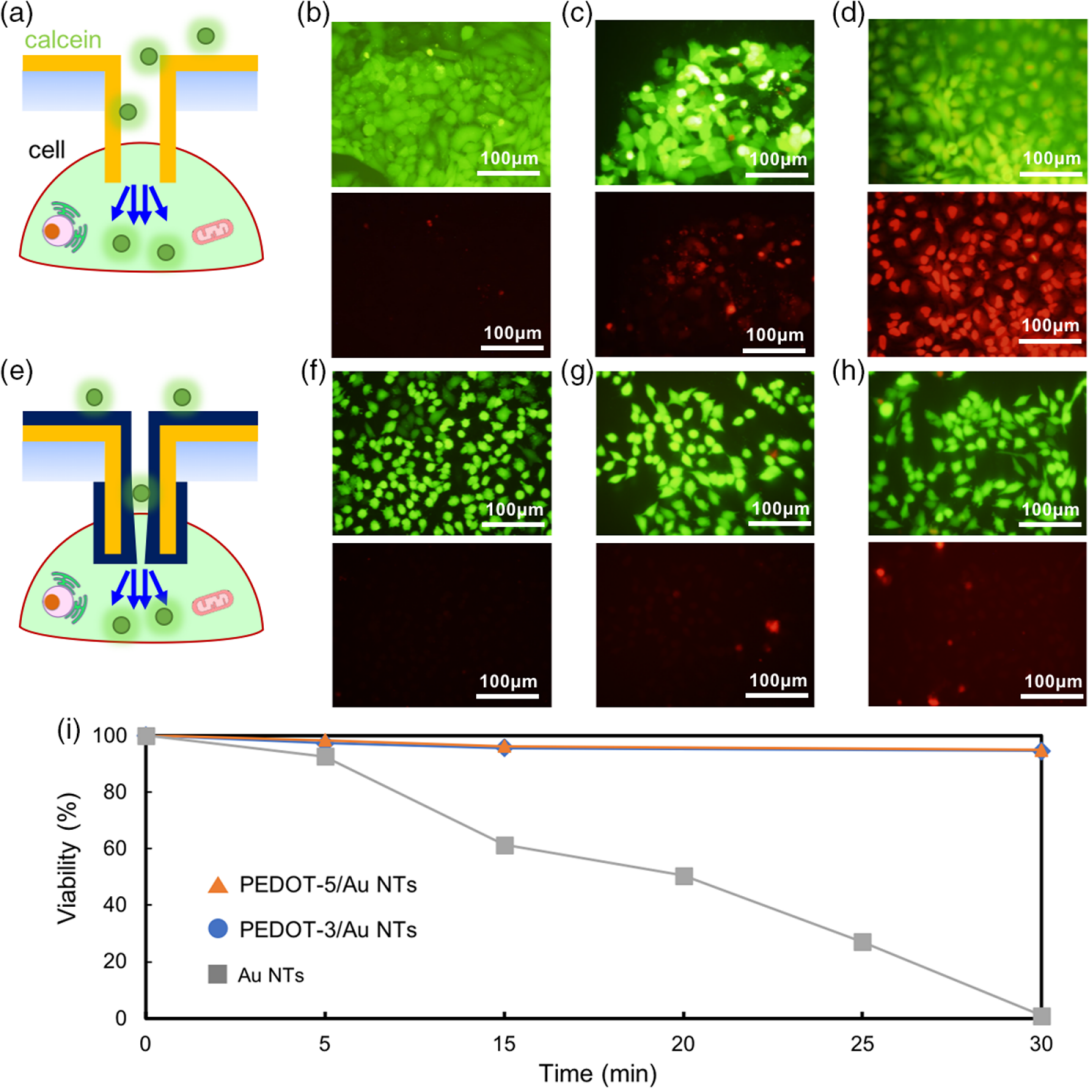
Cell viability after the stamping with different NTs. a) Schematic of Au NT stamping into HeLa cells. b–d) Fluorescence images of the calcein‐delivered cells (upper) with Au NT stamp at b) 5 min, c) 15 min, and d) 30 min. The cells were stained with PI to measure the dead cells (lower). e) Schematic of PEDOT/Au hybrid NT stamping. f–h) Fluorescence images of the calcein‐delivered cells (upper) with PEDOT‐3/Au NT stamp at f) 5 min, g) 15 min, and h) 30 min. The cells were also stained with PI for dead cell measurement (lower). i) Cell viabilities after the insertion of using various NT stamps. *n* = 197 cells with Au stamp, 194 cells with PEDOT‐3/Au stamp, and 178 cells with PEDOT‐5/Au stamp.

We examined the stamping of the hybrid NT membrane with an optical microscope combined with a manipulator to calibrate the insertion of the NTs into adhesive cells. In the same manner with the flux measurement through NT membrane, we fabricated three types of the NT stamps: 1) Au NTs (diameter: 750 nm, density: 2.2 × 10^7^ tubes cm^−2^, height: 2.4 μm), 2) PEDOT/Au NTs (referred as PEDOT‐3/Au, 3 min PEDOT coating, diameter: 269 nm, density: 2.2 × 10^7^ tubes cm^−2^, height: 2.4 μm), 3) PEDOT/Au NTs (referred as PEDOT‐5/Au, 5 min PEDOT coating, diameter: 123 nm, density: 2.2 × 10^7^ tubes cm^−2^, height: 2.4 μm). As in our previous paper,^[^ ^32^ ^]^ we used HeLa cancer cells to evaluate the viability after insertion. The viability (%) is defined as viability = live cells/live and dead cells, where live cells are the number of calcein‐stained cells, and dead cells are the number of propidium iodide (PI)‐stained cells. Furthermore, we confirmed the high viability of over 90% after inserting Au NTs into NIH‐3T3 cells for 5 min^[^ ^32^ ^]^ using this evaluation method. We can also obtain high viability of 92.6% after Au NT insertion into HeLa cells for 5 min (**Figure** **4**a,b). However, the viability decreased dramatically with an increase in the Au NT insertion time (Figure 4c,d), and over 99% of cells eventually died after 30 min of insertion. Consequently, the intracellular content is extracted from cells to the stamp chamber. In contrast, the insertion of the hybrid NTs into HeLa cells is shown in Figure 4e–h. The PEDOT layer coated on Au NTs acts as a spatial and charged barrier to suppress the extraction (molecular flow rate), as shown in Figure 3a. Thus, the coated Au NTs could exhibit better biocompatibility than the uncoated ones during physical insertion and long‐term contact with the living cells. Both PEDOT‐3/Au and PEDOT‐5/Au NT stamps can deliver calcein molecules into HeLa cells for 5 min of insertion with high viability of over 98%. Even when we inserted the hybrid NTs for 30 min, 94% cell viability remained (Figure 4i).

### 2.4 Electrochemical Control of Intracellular Delivery

**Figure 5:**
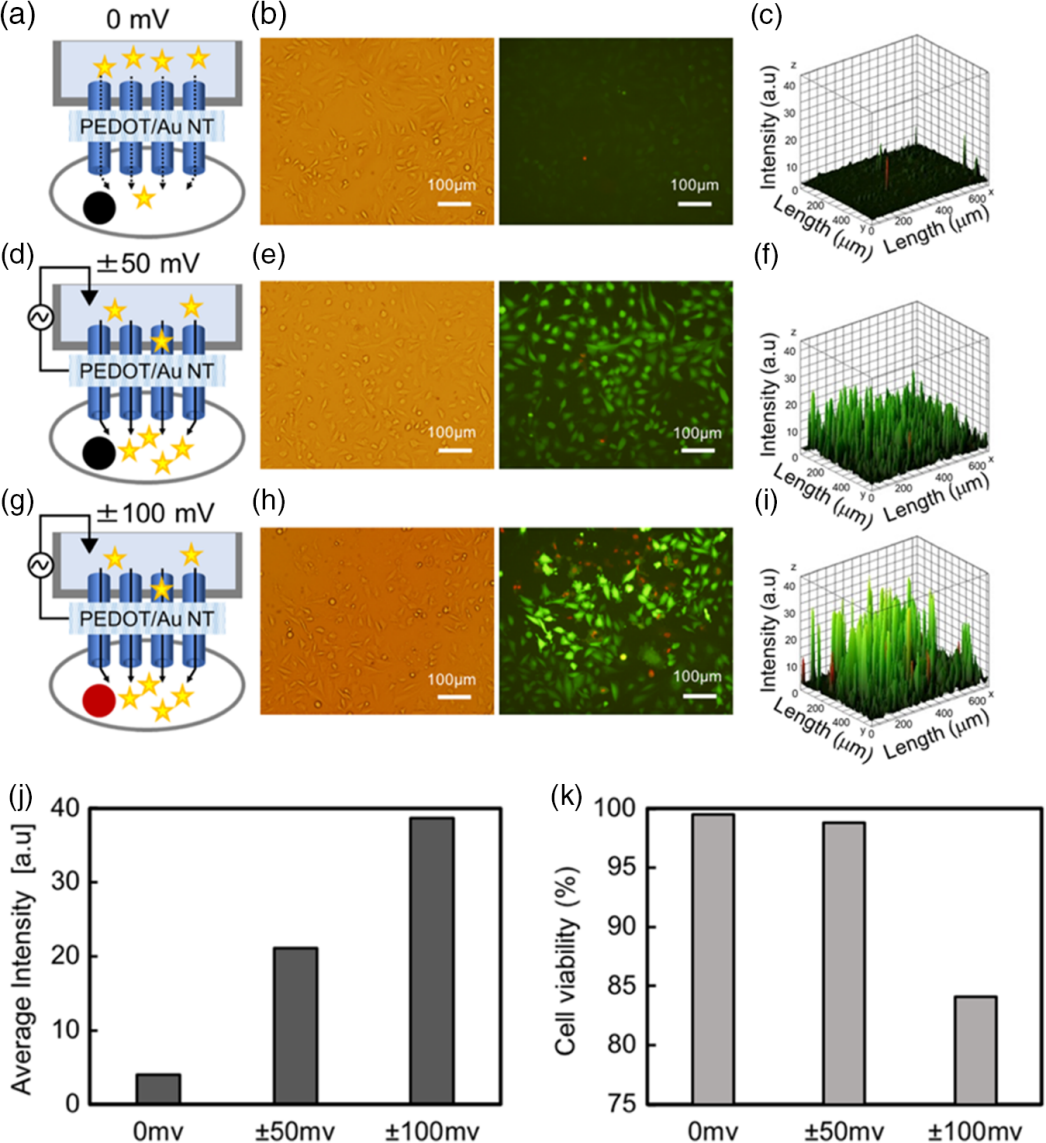
Electrical control of intracellular delivery. a) Schematic of PEDOT‐5/Au NT stamping into HeLa cells without the voltage. b) Optical (left) and fluorescence images (right) of the calcein‐delivered cells with PI treatment after the insertion for 10 min. c) The fluorescence intensity profile from the image (b). d) Schematic of PEDOT‐5/Au NT stamping with the applied voltage of ±50 mV. e) Optical (left) and fluorescence images (right) of the calcein‐delivered cells with PI treatment after the insertion with ±50 mV for 10 min. f) The fluorescence intensity profile from the image (e). g) Schematic of PEDOT‐5/Au NT stamping with the applied voltage of ±100 mV. h) Optical (left) and fluorescence images (right) of the calcein‐delivered cells with PI treatment after the insertion with ±100 mV for 10 min. i) The fluorescence intensity profile from the image (h). j) Average fluorescence intensity from the calcein molecules in the cells (*n* = 229) delivered by the different voltages. k) Cell viability after the stamping with the different voltages. *n* = 229.

To accelerate molecular delivery into the cells through the hybrid NTs, we demonstrate the calcein delivery into HeLa cells with the external voltage. Because the flux rate through the PEDOT‐5/Au NT without the voltage was around 9.82 × 10^−3^ nmol s^−1^ cm^−2^, the amount of the delivered calcein into the cells for 10 min is extremely low (**Figure** **5**a–c). In Figure 5, we used the exposure time of 1 s to obtain the fluorescence images. The average fluorescence intensity (a.u.) from the calcein‐stained cells (*n* = 229) is 4.0, and its viability is 99.5%. The intensity enhanced to 21 by applying an AC voltage of ±50 mV (Figure 5d–f,j) and 38.7 by ±100 mV (Figure 5g–j). However, the viability decreased gradually to 96.9% with ±50 mV and 84.1% with ±100 mV (Figure 5k). The viability of the calcein‐injected cells remained at 98.9 and 98.7% at the DC voltage of 50 and −50 mV, respectively (Figure S6, Supporting Information); however, the average fluorescence intensities from the stained cells were lower than those when AC voltage was applied owing to the low flow rate of calcein molecules through the hybrid NTs (Figure 3c). There was a small difference in calcein flow in the PBS solution and adhesive cells. This is because the ion concentration and viscosity in PBS were lower than those in the cell. Furthermore, the cells can release calcein molecules via exocytosis process; hence, the measurements were distributed by cell activities. Nevertheless, compared with conventional hybrid methods such as the combination of nanostructures with an electric^[^ ^23^ ^]^ and photothermic poration,^[^ 24, 25 ^]^ present electrochemical hybrid NT stamping provides high viability of over 96% by applying a small voltage of ±50 mV, which is less than common membrane potentials of about 70 mV.^[^ ^39^ ^]^ Furthermore, the hybrid NTs accelerate calcein delivery into the cells and control the flow rate with the small external voltage.

**Figure 6:**
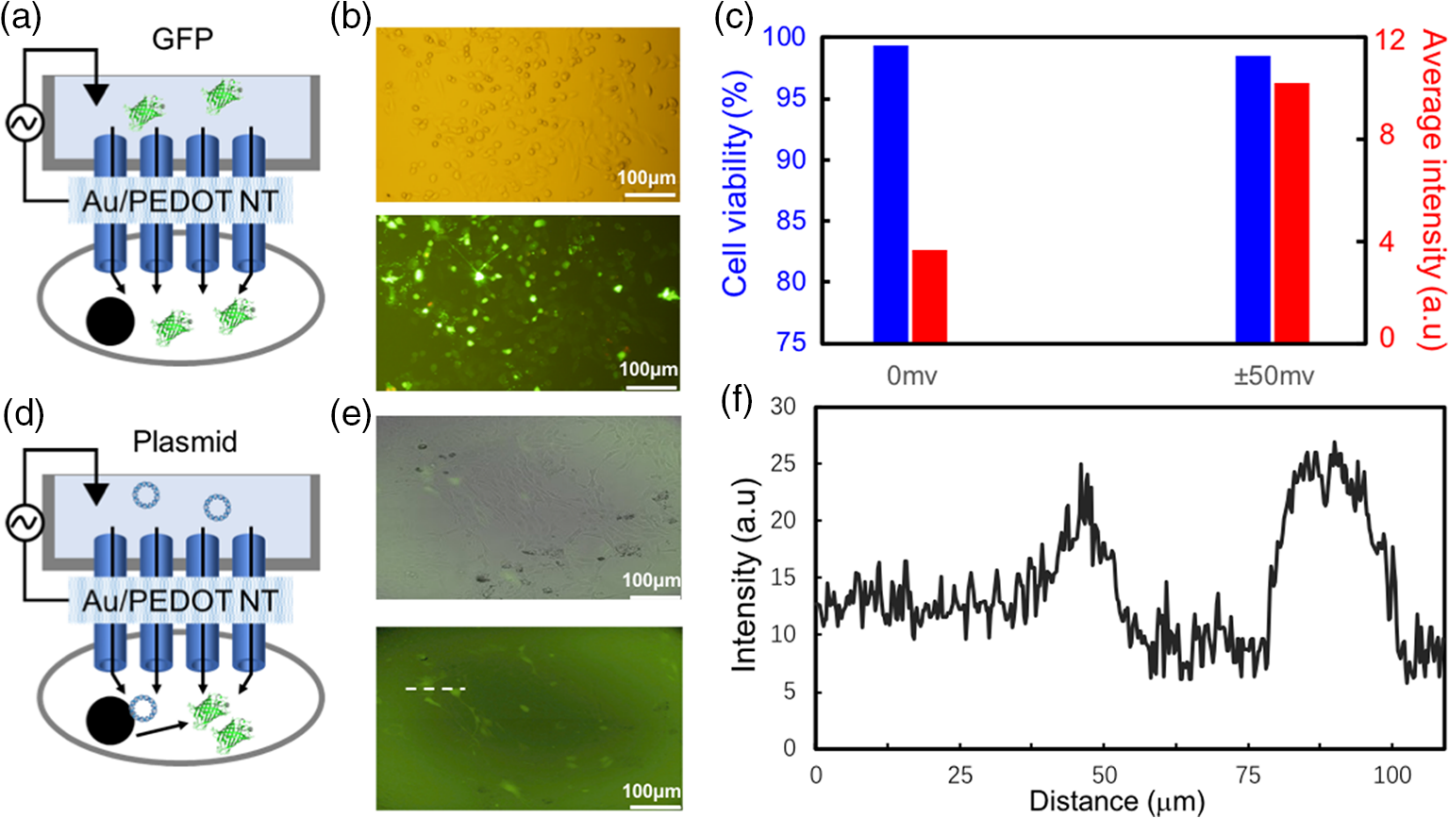
Macromolecule delivery into the different types of adhesive cells. a) Schematic of PEDOT‐5/Au NT stamping for GFP protein delivery into HeLa cells with the voltage supply. b) Optical (upper) and fluorescence images (lower) of the GFP‐delivered cells after the stamping with ±50 mV supply for 10 min. c) The cell viability and average fluorescence intensity after the stamping with the different voltages. *n* = 241. d) Schematic of PEDOT‐3/Au NT stamping for GFP plasmid gene delivery into NIH‐3T3 cells with the voltage supply of ±50 mV for 10 min. e) Optical (upper) and fluorescence images (lower) of the GFP‐expressed cells after culturing for 2 days. f) The fluorescence intensity profile of the image regions (e) marked by the dashed lines.

Moreover, the hybrid NT stamp is applied to deliver the large molecules of GFP into HeLa cells (**Figure** **6**a–c) and the GFP plasmid into NIH‐3T3 cells (Figure 6d–f). The hybrid stamp consists of the PEDOT‐5Au NT membrane and glass tube, including 0.8 mm GFP in pH7.4 HEPES buffer. We stamped the hybrid NTs into the cells for 10 min. After the stamping, the average fluorescence intensity from the GFP delivered into HeLa cells is 3.6 with 99.3% viability. As the GFP protein is a large molecule (25 kDa) and its *J* of 0.179 nmol s^−1^ cm^−2^ (Figure S7, Supporting Information) is smaller than the calcein molecule (0.361 nmol s^−1^ cm^−2^, Figure 3a), the GFP delivery into cells is slow without the applied voltage. However, the GFP delivery with the hybrid stamp could accelerate when we applied ±50 mV. The intensity from the injected GFP enhanced to 10.2 with the remaining high viability (98.5%). The delivery efficiency of GFP protein is 84%, which is high enough compared with conventional methods.^[^ ^40^ ^]^

Similar to GFP protein experiments, we investigate the delivery of the GFP plasmid gene into NIH‐3T3 using the hybrid NT stamp (Figure 6d–f). The hybrid stamp consists of the PEDOT‐3/Au NT membrane and glass tube, including 10 mg L^−1^ GFP plasmid in 2 mL Opti‐MEM. We carried out the plasmid delivery with the applied voltage of ±50 mV to the hybrid stamp for 10 min when we insert the stamp into the cells (Figure 6d). After the delivery, we cultured the cell in a CO_2_ incubator to express the GFP inside the cells. After 2 days, we could observe the GFP‐expressed cells with a fluorescence microscope (Figure 6e). The transfection rate using the hybrid NTs is about 10%, significantly higher than with endocytosis (less than 1%, Figure S8a,b, Supporting Information). As compared with conventional methods,^[^ ^17^ ^]^ this value is high enough. As a controlled experiment, we demonstrate the plasmid delivery without the applied voltage, resulting in no GFP expression in the cells (Figure S8c,d, Supporting Information).

## 3 Conclusion

We have developed the PEDOT/Au hybrid NT stamping with high efficient and precise dosage control by the small external voltage. The hybrid stamp improved the cell viability by over 94% for 30 min physical insertion while decreased to less than 1% using original Au NTs. Furthermore, the hybrid stamp acted as an electrochemical gate that can open the pore at ±50 mV to transport small molecules of calcein dye with high efficiency (99%) and high viability (96.8%). Also, the hybrid nanogate can transport large molecules both of a GFP protein with 84% efficiency and 98.5% viability and of a GFP plasmid at a transfection rate of approximately 10%. Thus, the present hybrid stamping can potentially deliver versatile molecules into adhesive cells with high efficiency and viability.

## 4 Experimental Section

4.1

4.1.1

##### Fabrication of the PEDOT/Au NT Membrane

Following our previous work,^[^ ^32^ ^]^ we coated a tin–palladium metallic catalyst layer on the TEPC surface to form an Au film on the TEPC membrane (it4ip S.A.). Then, we immersed the membrane in an Au‐plating solution (NC Gold II, Kojima Chemicals) at 40 °C overnight to obtain the Au/TEPC membrane. The top surface of Au/TEPC membrane was etched with aqua regia (ITO‐02, Kanto Chemical Co.) for removing Au nanolayer and subsequent O_2_ plasma for etching TEPC to control the height of Au NT/TEPC membrane. The Au NT/TEPC membrane was immersed into a solution containing 0.05 m EDOT and 0.1 m LiClO_4_, and then the voltage of 1 V was applied to the membrane to form the PEDOT on Au NTs. After the polymerization, the PEDOT/Au NT hybrid membrane was rinsed with ethanol and water to remove residual EDOT monomer from the membrane and then dried and stored it in a vacuum chamber. Before using the membrane, we confirmed the geometry of PEDOT/Au NTs with a scanning electron microscope (S‐3400N, HITACHI SEM).

##### Flux Measurements

For the measurements, we made a needle stamp for the source chamber that consists of the hybrid membrane (diameter: 8 mm) of PEDOT/Au NTs at different coating times and 10 mm PBS solution (pH 7.4), including 1.6 mm calcein molecules in a glass tube, and then contacted it to the collection chamber including the stirring PBS solution. After that, the calcein molecules diffuse from high‐concentrated source chamber to the collection chamber through the PEDOT/Au hybrid NT membrane. Then we connected the PEDOT/Au NTs working electrode, the Pt counter electrode, and the Ag/AgCl reference electrode to the electrochemical power supply (HA‐151B and HB‐305, Hokuto Denko) to apply the voltage to a needle stamp. Next, we measured the amount of the transported calcein with and without the applied voltage by the microplate reader (Fluoroskan FL, Thermo Fisher Scientific). The solution was collected every 10 min for the measurement.

##### Cell Culture

We used NIH 3T3 cells (RCB2767; National Research and Development Corporation RIKEN BioResource Research Center, Japan) and HeLa cells (RCB0007; National Research and Development Corporation RIKEN BioResource Research Center, Japan) as adhesive cells. We cultured the cells in Dulbecco's modified eagle medium (12 800 017, Gibco| Thermo Fisher Scientific, USA) supplemented with 10% fetal bovine serum (26 140 079, Gibco| Thermo Fisher Scientific, USA), 0.0588 g L^−1^ penicillin G potassium (P7794, Sigma‐Aldrich), and 0.1 g L^−1^ streptomycin sulfate salt (S6501, Sigma‐Aldrich). In addition, we used 0.5% trypsin (T4049, Sigma‐Aldrich) to obtain the cell suspension and disperse the cell suspension on the 2.5 cm cell culture dish. Finally, we culture the cells in a 5% CO_2_ incubator at 37 °C.

##### NT Stamping into the Cells

We used an inverted optical microscope (IX71 or IX83, Olympus) combined with an *x*–*y*–*z* manipulator for hybrid NT stamping into the cells, as described in our previous work.^[^ ^32^ ^]^ The NT stamp was inserted into the target cells with a manipulator and observed using differential interference contrast (DIC) (Figure S9, Supporting Information). First, we focused at the bottom and top of the adhesive cells to evaluate the average height of the cells (Figure S9a, Supporting Information). Thereafter, by lowering the focus by a few micrometers from the position at which the top of the cell was focused, the focus became blur. This position corresponds to the insertion depth of the NTs, and when the needle was lowered to this position, it focused at the tip of the NTs (Figure S9b, Supporting Information). From the cell images in Figure S9a,c, Supporting Information, we measured the average area of the adhesive cells (91 ± 23 μm^2^). As the density of NTs is 2.2 × 10^7^ tubes cm^−2^, 20 ± 5 NTs will be inserted into one cell at a time. After removing the NTs from the cells, we could observe the stained cells using a fluorescence microscope (Figure S9c, Supporting Information). Different stamp types were utilized, including 1.6 mm calcein (MP Biomedicals, Inc., molecular weight [MW]: 622.5 g mol^−1^) for calcein delivery, 0.8 mm GFP (MW: 25 kDa) for GFP delivery, and 10 mg L^−1^ GFP plasmid (pAcGFP1‐C1, Takara Bio., MW: 4.7 kDa) for plasmid delivery, respectively. The GFP protein was expressed by a cell‐free protein synthesis system as described in the previous works.^[^ ^41^ ^]^ To confirm the dead cells, we stained them with a PI indicator for 30 min.

## Conflict of Interest

The authors declare no conflict of interest.

## Data Availability Statement

Research data are not shared.

## Supporting information

Supplementary Material
