# Supplementary material for: High‐Efficient and Dosage‐Controllable Intracellular Cargo Delivery through Electrochemical Metal–Organic Hybrid Nanogates
Source: Small Sci. 2021 Sep 9;1(12):2100069. doi: 10.1002/smsc.202100069 (PMC11936059; doi:10.1002/smsc.202100069)
Supplement: Supplementary file 1 — Supplementary Material [file SMSC-1-2100069-s001.pdf]

Supporting Information

Title: Highly Efficient and Dosage-Controllable Intracellular Cargo Delivery through Electrochemical Metal-Organic Hybrid Nanogates

Bowen Zhang, Dinuo Zheng, Shi Yiming, Kazuhiro Oyama, Masahiro Ito, Masaomi Ikari, Takanori Kigawa,Tsutomu Mikawa, Takeo Miyake

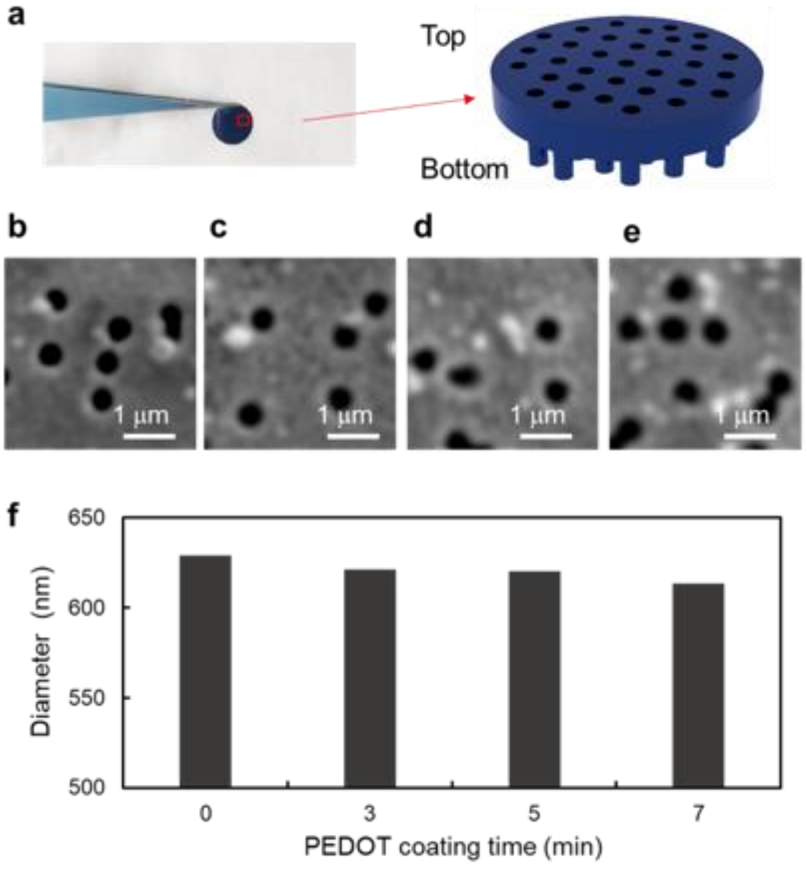

Figure S1. a) Image and schematics of PEDOT/Au membrane on the top surface. b–e) SEM images of the top surface of Au NTs at different PEDOT film-coating times of (b) 0, (c) 3, (d) 5, and (e) 7 min. f) Diameter of PEDOT/Au membrane on the top surface.

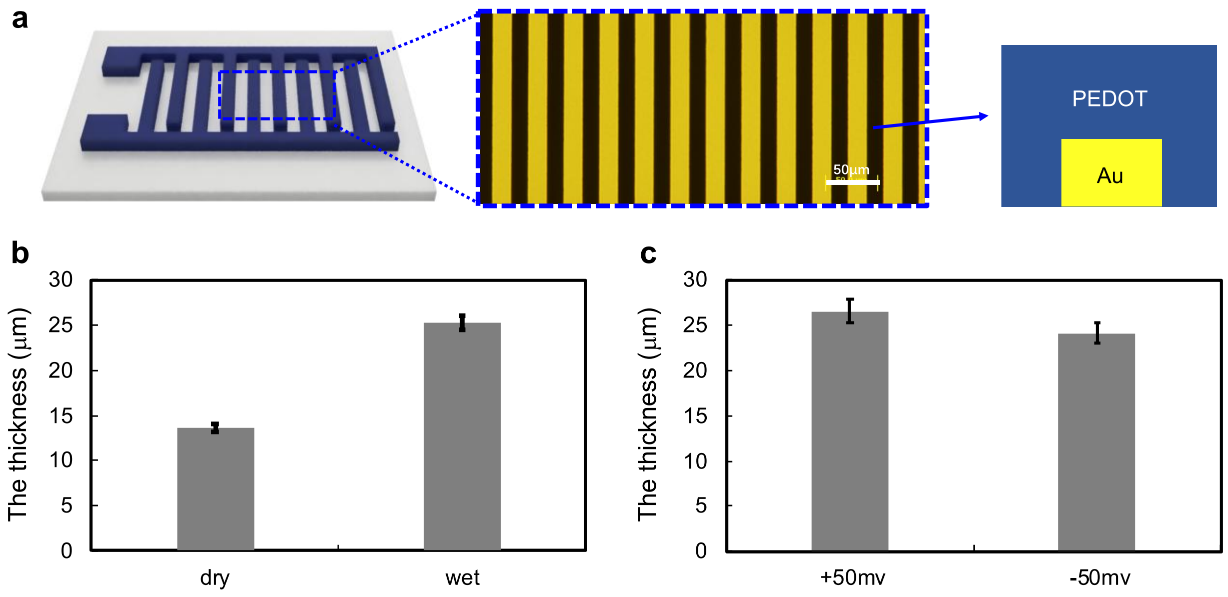

Figure S2. a) Image and schematics of PEDOT/Au comb electrode. b) Thickness of PEDOT/Au measured using a laser microscope in dry and wet conditions. c) Thickness of PEDOT/Au measured using a laser microscope when we applied different voltages to the PEDOT/Au in pH 7.4 PBS.

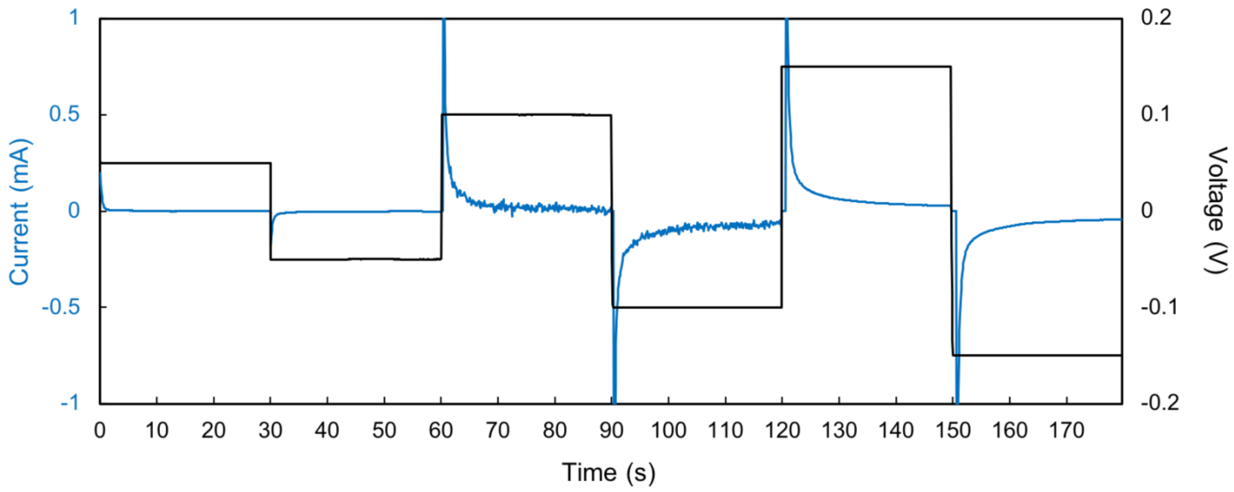

Figure S3. Current measurements of the PEDOT-5/Au NT electrode at different voltages of 50, −50, 100, −100, 150, and −150 mV in a PBS solution with a pH = 7.4.

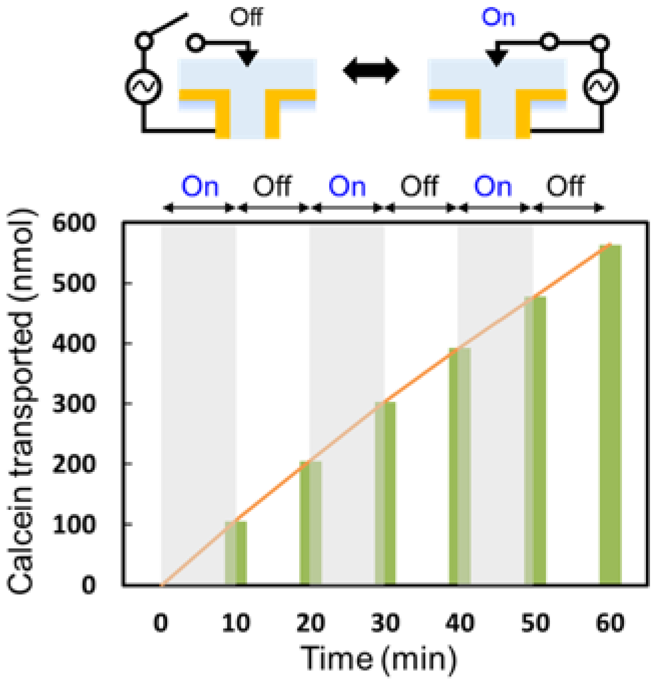

Figure S4. Amount of transported calcein through Au NTs with applied sequential voltages of ±50 and 0 V for three cycles.

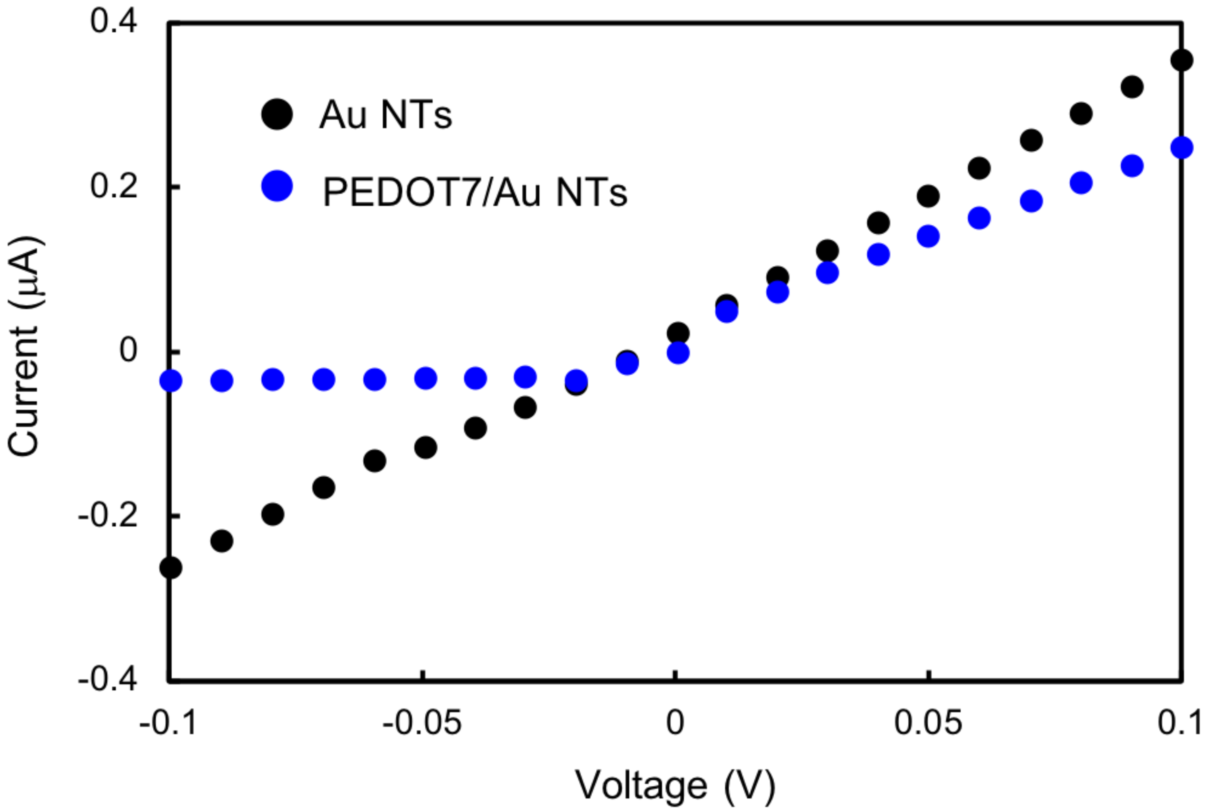

Figure S5. Ionic current through Au and PEDOT7/Au NTs at different voltages.

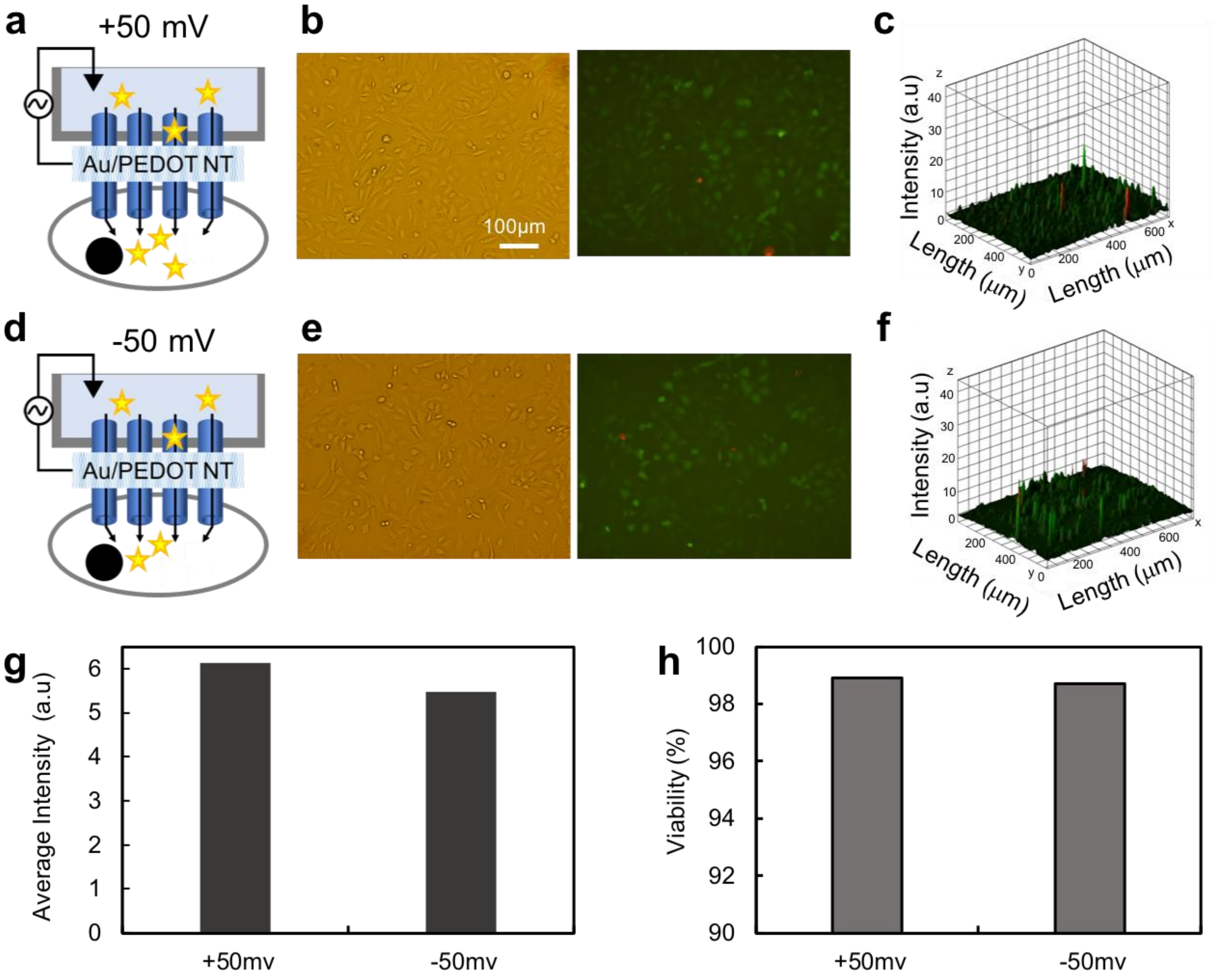

Figure S6. a) Schematic of PEDOT-5/Au NT stamping into HeLa cells with a 50-mV supply. b) Optical (left) and fluorescence (right) images of the calcein-delivered cells with PI treatment after the insertion for 10 min. c) Fluorescence intensity profile from (b). d) Schematic of PEDOT-5/Au NT stamping with an applied voltage of −50 mV. e) Optical (left) and fluorescence(right) images of calcein-delivered cells with PI treatment after the insertion at −50 mV for 10 min. f) Fluorescence intensity profile from (e). g) Average fluorescence intensity of calcein molecules in the cells (n = 250) delivered by the different voltages. h) Cell viability after stamping at different voltages (n = 250).

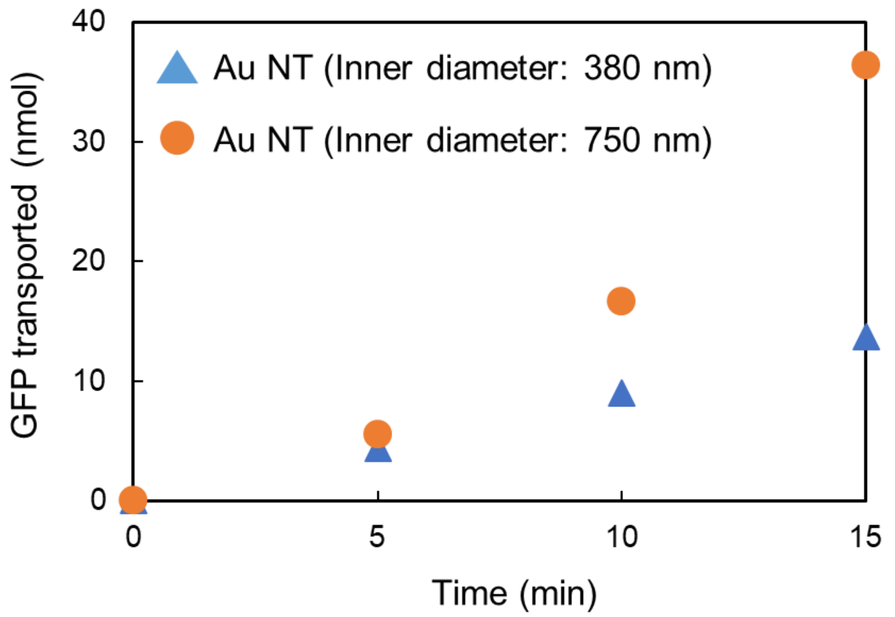

Figure S7. Amount of transported GFP through Au NTs with the inner diameters of 380 and 750 nm.

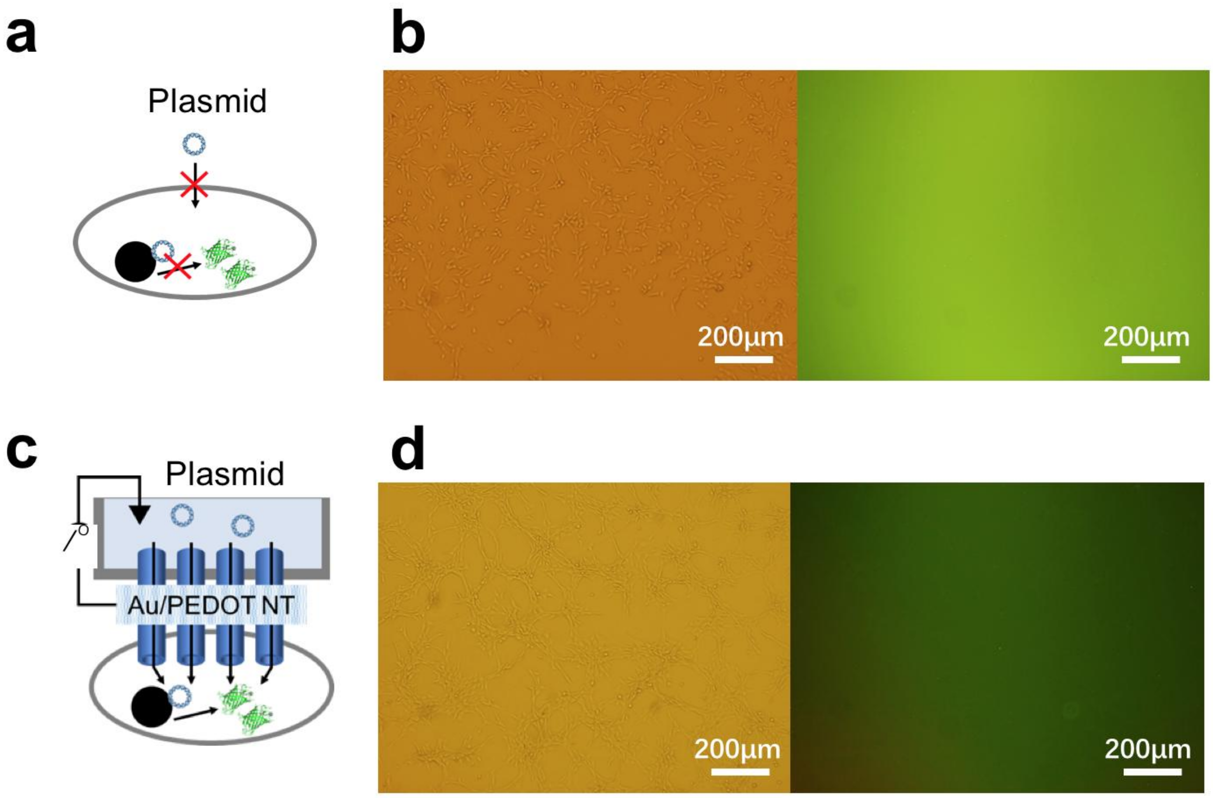

Figure S8. a) Schematic of co-incubation of NIH-3T3 cells and GFP plasmid for 2 days in a 5% CO_2 incubator at 37 °C. b) Optical (left) and fluorescence (right) images of the co-incubated cells. c) Schematic of PEDOT-3/Au NT stamping for GFP plasmid gene delivery into NIH-3T3 cells for 10 min without voltage supply. d) Optical (left) and fluorescence (right) images of stamped cells after culturing for 2 days.

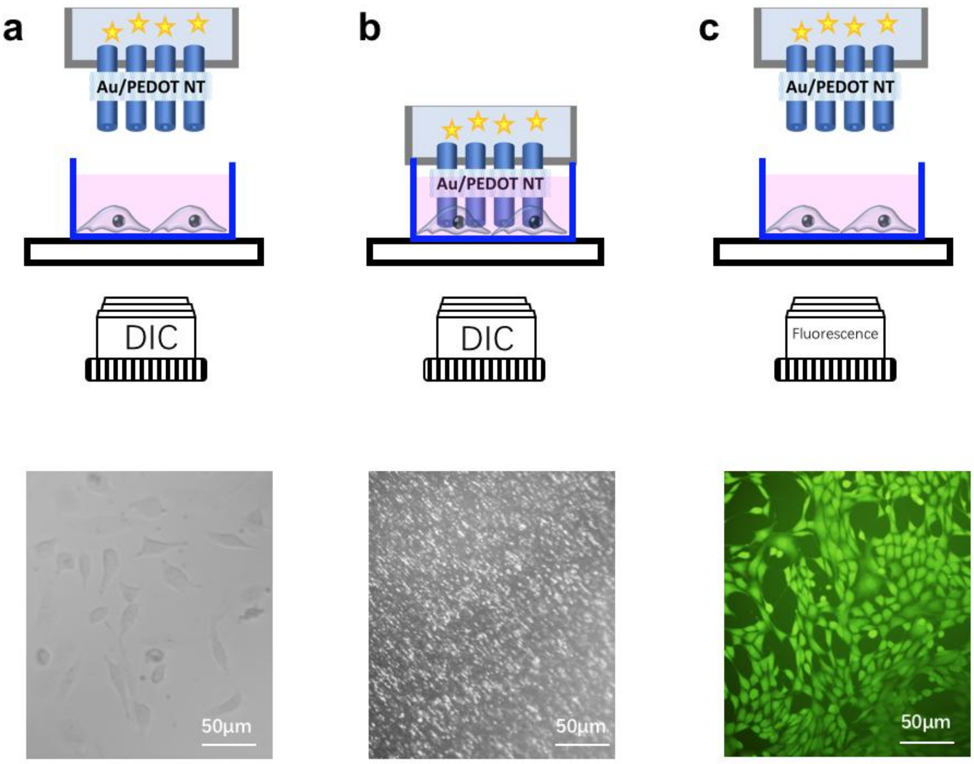

Figure S9. Schematic and images of our stamping system. a) Cell image obtained using a DIC microscope to evaluate the average height of adhesive cells. b) NT image at the insertion depth controlled by the DIC microscope. c) Image of the stained cells after injection (obtained using a fluorescence microscope).
